# Intra- and inter-limb strength imbalance and asymmetry in soccer: A comparison of elite senior and junior players

**DOI:** 10.1371/journal.pone.0302474

**Published:** 2024-04-26

**Authors:** Robert Śliwowski, Thierry Paillard, Łukasz Bojkowski, Witold Dudziński, Mikołaj Patek, Jakub Marynowicz

**Affiliations:** 1 Department of Theory and Methodology of Team Sport Games, Poznan University of Physical Education, Poznan, Poland; 2 Department of Sport Sciences, University of Pau et des Pays de l’Adour, E2S UPPA, MEPS Laboratory, Tarbes, France; 3 Department of Psychology, Poznan University of Physical Education, Poznan, Poland; 4 Rehasport Clinic FIFA Medical Centre of Excellence, Poznan, Poland; Università degli Studi di Milano: Universita degli Studi di Milano, ITALY

## Abstract

Evaluation of muscle strength imbalance can be an important element in optimizing the training process of soccer players. The purpose of the study was to examine isokinetic peak torque (PT) and total work (TW) exerted by both knee extensors (quadriceps or Q) and flexors (hamstrings or H), intra-limb imbalance and the magnitude and direction of inter-limb asymmetry in top elite senior (n = 109) and junior (n = 74) soccer players. An isokinetic dynamometry was used to measure maximum peak torque of quadriceps (PT-Q) and hamstrings (PT-H) at an angular velocity of 60° ·s^-1^, as well as the total work for extensors (TW-Q) and flexors (TW-H) at an angular velocity of 240° ·s^-1^ in the dominant (DL) and non-dominant leg (NDL) during concentric muscle contraction. Intra-limb imbalance and inter-limb asymmetries were calculated using a standard equation. Statistical analysis using t-test and Mann-Whitney U-test revealed: (a) no differences (*p* > 0.05) between groups for PT-Q and PT-H, (b) greater strength levels (*p* < 0.05) for TW-Q and TW-H of senior players than juniors, and (c) no differences (*p* > 0.05) between groups for intra-limb imbalance and inter-limb asymmetry. Additionally, Pearson’s chi-kwadrat (χ2) analysis showed no differences (*p* > 0.05) between groups for intra-limb imbalance and inter-limb asymmetry in relation to the ’normative’ values accepted in the literature that indicate an increase in the risk of knee injury. This study shows that isokinetic assessment can be an important tool to identify imbalances/asymmetries and to develop strategies to reduce the risk of muscle injury.

## Introduction

In modern soccer, every action and context of the game takes place at ever-faster speeds, in ever-smaller playing areas, with ever-more players. This requires soccer players to be able to accelerate quickly, stop, change direction, sprint and sprint repeatedly over long periods [1–6]. All these requirements call for strength and power in the lower limbs [1,7] which in turn calls for increasingly precise neuromuscular assessment and preparation of soccer players by coaches and their staff [8]. To this end, the isokinetic dynamometer has often been recognized as the gold standard for measuring strength [9,10].

An isokinetic dynamometer also enables reliable assessment of the muscular strength imbalance of the lower limbs, both bilateral (inter-limb asymmetry) and unilateral (intra-limb imbalance or flexor-extensor muscles ratio or intra-limb ratio) [4,11–15]. Muscle strength asymmetries may negatively affect specific performance [3,16–19], and soccer-related abilities [20,21], thus significantly limiting the players’ sporting performance [14,18,21]. In addition, muscle strength imbalance and asymmetry is a particularly important predictor of many different injuries in soccer players [2,11,12,14,15,22–25]. Furthermore, imbalances and asymmetries in muscle strength are also relevant factors in determining the return to play process after a therapeutic program following injury and rehabilitation [11,22,26].

A number of factors have been identified as facilitating and limiting the presence of imbalance and asymmetry in soccer players. One of the factors facilitating asymmetry is training time, and research shows that the longer the training time, the greater the asymmetry, particularly in professional soccer players [27,28]. By contrast, one the factors limiting asymmetry is the age of athletes. Despite their greater experience, older players showed lower strength asymmetry and higher strength indexes compared to young athletes [12,24,29,30]. Experienced soccer players can also develop neuromuscular properties and preventive strategies that can limit fatigue and injury, and facilitate resilience [24,31]. Young soccer players accounted for a higher proportion of muscle strength imbalances than their senior compatriots [12]. Moreover, Bishop et al. [13] demonstrated higher intra-limb ratio values at an angular velocity of 60°·s^-1^ and lower inter-limb asymmetry values at angular velocities of both 60° ·s^-1^ and 300° ·s^-1^ (except for hamstrings at slow speed) in professional soccer players compared to younger academy players. By contrast, some authors did not confirm any significant effect of age on level of asymmetry [4,25,32]. Taken together, the results mentioned above show that there is a lack of evidence surrounding muscle strength imbalance and asymmetry differences between senior and youth soccer players playing at the highest level of competition. In particular, it is currently unknown whether the extent of imbalance and asymmetry differs between elite adult players and younger ones, who have had markedly less exposure to the specific asymmetric movement patterns that are part and parcel of elite professional soccer players. Yet, a better understanding of the impact and risk of imbalances and asymmetries would help to refine the profiles of soccer players in terms of performance potential and injury prevention, and thus offer practical insights into how to optimize senior and junior training development.

Hence, the purpose of this study is twofold: (1) to assess the isokinetic strength exerted by both knee extensor and flexor muscles in top elite senior and junior soccer players and; (2) to identify the nature, and quantify the level of intra- and inter-limb strength imbalances and asymmetries between both these groups. We hypothesized that (1) senior players will display greater isokinetic strength exerted by both knee extensor and flexor muscles than junior soccer players; and (2) junior soccer players will demonstrate greater asymmetries and/or imbalances than senior players.

## Materials and methods

### Participants and data collection

The cohort for this study included 109 male senior (S) group and 74 male junior (J) group soccer players. The senior players (age: 25.1 ± 4.3 years; height: 182.4 ± 6.1 cm; body mass: 78.1 ± 7.3 kg; training experience: 16.3 years) included international (mainly Poland and countries of Southern, Eastern and Northern Europe) elite players from the Polish Ekstraklasa (the top competition level in Poland). All the participants from the senior group had at least three years of playing experience at a professional level and participated in regular training sessions (as outlined in their contracts). The junior group (age: 17.2 ± 1.5 years; height: 180.6 ± 7.4 cm; body mass: 72.3 ± 8.4 kg; training experience: 9.5 years) were soccer players from the leading soccer academy in Poland and had all been active in high-level soccer competitions. Over 50% of the surveyed juniors represented Poland in particular age categories and over the period of data collection many won national junior titles. The study was conducted using retrospective clinical data from professional soccer players, collected between 2010 and 2022. The data was retrieved for research purposes between May 4 and May 31 2022, during and after which the authors had no access to information that would allow for identification of individual participants. All measurements were performed twice per year. The first measurements were taken in January/February (during the league season’s winter break in Poland), whilst the second were taken during pre-season training in June/July, with the season officially starting in August each year. Each season, every participant performed two functional movement screen tests, isokinetic strength tests for hamstring, quadriceps and knee, proprioception tests, and ground reaction force tests. All of these tests were part of routine biomechanical evaluations. An annex in their professional contracts informed players about the risks of the tests. All participants provided written consent for their data to be collected and used. For players aged under 18, parents or guardians were informed about the risks and their written, informed consent was obtained before their ward participated in the study. The study was conducted in accordance with the requirements of the Declaration of Helsinki and ethical approval for the study was granted by the Bioethics Committee at Poznań University of Medical Sciences (629/13).

### Lower-limb muscle strength testing

The lower limb muscle strength assessments were conducted at the Rehasport Clinic, recognized as a FIFA Medical Centre of Excellence, in Poznan, Poland. All measurements were carried out by the same team of researchers. Knee muscle strength was evaluated using the Biodex System 3 dynamometer (Biodex Corp, Shirley, NY, USA), measuring peak torque (PT) and total work (TW). High test-retest reliability was observed (intra-class correlation coefficient [ICC] = 0.93–0.95) [33]. Procedures concerning the alignment of participants’ dynamometer rotation axis, positioning, gravity correction, and stabilization adhered to established guidelines outlined in the literature [34,35]. Prior to the isokinetic assessment, each participant underwent a 10–15-minute warm-up session, including pedaling on a Monark 828E Ergomedic stationary cycle ergometer (Monark, Vansbro, Sweden) at a moderate pace (50–100 watts) and dynamic stretches targeting major lower limb muscle groups [34,36]. Isokinetic torque of the quadriceps and hamstring muscles was assessed through continuous, bidirectional knee extension-flexion movements at angular velocities of 60° ·s^-1^ and 240° ·s^-1^, spanning a range of motion from 0° (flexion) to 90° (full extension). Testing at angular velocities of 60° ·s^-1^ and 240° ·s^-1^ has been widely employed in previous studies investigating muscle strength in soccer players [11,12,14,19]. Participants underwent three trials at submaximal efforts, gradually increasing the load to 50%, 75%, and approximately 100% of their maximum capability, followed by one set of three repetitions at maximal concentric contraction at an angular velocity of 60° ·s^-1^ and 30 repetitions at an angular velocity of 240° ·s^-1^. The same protocol was repeated for the opposite leg. Participants were given a 30-second rest after the third submaximal trial, followed by a one-minute break between angular velocities, and a three-minute break during which machine settings were adjusted for the opposite leg. Participants were instructed to complete the movement through the full range of motion. The order of testing for dominant (DL) and non-dominant (NDL) legs was randomized [34,35]. Dominance was determined based on participants’ preferences when kicking a ball [34]. Only windowed data was analyzed to focus on constant velocity periods. Statistical analysis included relative PTs (normalized by body weight and expressed in Nm·kg^-1^) for flexors (PT-Q) and extensors (PT-H) in both legs, unilateral muscle torque ratios for dominant and non-dominant extremities (HDL/QDL and HNDL/QNDL, respectively) at an angular velocity of 60° ·s^-1^, and relative TWs (J·kg^-1^) for extensors (TW-Q) and flexors (TW-H) for both legs at an angular velocity of 240° ·s^-1^. All tests were conducted before 1 pm to minimize inter-day variability and were performed in the same sequence for each participant. Participants refrained from intensive training for 48 hours preceding the testing. Before commencing the test, participants completed a questionnaire regarding any musculoskeletal pain, discomfort, or known lower extremity injuries. Participants with major or moderate lower leg, knee or thigh injuries were excluded from further analysis. None of the participants had a history of significant knee injuries, anterior cruciate ligament (ACL) repairs or rehabilitation, leg fractures, or surgeries within the year preceding the evaluation.

### Intra-limb ratio and inter-limb asymmetry analysis

In accordance with the primary goal of the study, the intra-limb ratio was determined on the basis of the so-called conventional H/Q ratio (hamstring-to-quadriceps ratio), which is calculated by dividing the CON (concentric) PT-H by the concentric PT-Q of the same limb at an angular velocity at 60° ·s^-1^ [37]:

$$H/Q_{CON}ratio=PT-H_{CON}/PT-Q_{CON}.$$

Next, inter-limb asymmetries for all isokinetic test variables (PT-Q, PT-H, TW-Q and TW-H) were quantified as the percentage difference between the two limbs using the following equation [13,17]:

Asymmetry(%)=100/(maximumvalue)×(minimumvalue)×(−1)+100.

An ‘IF function’ was added to the end of the formula in Microsoft Excel to indicate inter-limb differences individually: *IF (non-dominant < dominant, 1, -1). This converted the asymmetry value to negative if the non-dominant kicking limb was stronger [17,19]. An intra-limb ratio below the cut-off value of 0.6 and inter-limb asymmetry below the cut-off value 10% respectively have previously been proposed as “normative values” to categorize soccer players as in the group at greater risk of knee injury. These “normative values” have also been considered an acceptable value for professional soccer players [15,26,36,38–40].

### Statistical analysis

All statistical analyses were performed using Statistica Version 13.0 (StatSoft Polska Sp. z o.o. 2020, Krakow, Poland). All of the examined quantitative variables (PT-Q, PT-H, H/Q ratio, TW-Q, and TW-H) are presented as means and standard-deviations. “Normative values" for all of these variables were also calculated on the basis of amounts and percentages to achieve cut-off points. The Shapiro-Wilk test was employed to ascertain the normality of the data distribution and Levene’s test was used to check homogeneity of variance. For all strength variables, depending on distribution of data, a *t*-test and Mann-Whitney U-test were conducted in order to calculate the difference between the results of the senior and junior groups. In order to indicate practical differences between groups, Hedge’s *g* effect sizes with 95% confidence intervals were calculated and interpreted as: trivial: <0.20, *small*: 0.20–0.50, *moderate*: 0.50–0.80 or *large*: >0.80 [41]. In turn, between-group differences for variables categorized as "normative values" were performed with the help of Pearson’s chi-kwadrat (χ2), which was used to compare the prevalence of intra-limb imbalance (≥0.6) and inter-limb asymmetry (>10%). The effect size was assessed using the phi coefficient, where values of 0.1, 0.3, and 0.5 are considered small, medium, and large effects, respectively. The relationship between intra-limb ratio and inter-limb asymmetries over the average of the two limbs for all variables were illustrated with scatter plots. Statistical significance was calculated as 0.05 for all statistical procedures.

## Results

Table 1 shows mean and SD data for all isokinetic strength measures. In general, the senior group showed greater strength levels for all variables for both legs (PT-Q, PT-H, TW-Q and TW-H), as represented by trivial to moderate ES values (*g* range = 0.03 to 0.55). Intergroup differences were found only for TW-Q and TW-H, both in the case of dominant and non-dominant leg. Intra-limb (H/Q) ratios showed small to trivial ES values (*g* range = 0.14 to 0.27) and did not differ between groups in both legs.

**Table 1:** Mean ± standard deviation (SD) strength data for both limbs, and Hedges g effect sizes (ES) with 95% confidence intervals (CI).

Variables	Senior Group n = 109	Junior Group n = 74	Hedges g (95% CI)	g Descriptor
**PT-Q** [Nm ·kg^-1^]				
Dominant	3.26 ± 0.39	3.25 ± 0.38	0.03 (-0.27, 0.32)	trivial
Non-Dominant	3.19 ± 0.43	3.15 ± 0.40	0.10 (-0.20, 0.39)	trivial
**PT-H** [Nm ·kg^-1^]				
Dominant	1.93 ± 0.31	1.86 ± 0.27	0.24 (-0.06, 0.53)	small
Non-Dominant	1.83 ± 0.28	1.79 ± 0.27	0.14 (-0.15, 0.44)	trivial
**TW-Q** [J ·kg^-1^]				
Dominant	51.49 ± 6.90	47.66 ± 6.96	**0.55 (0.25, 0.85)**	moderate
Non-Dominant	50.11 ± 6.33	47.29 ± 6.39	**0.44 (0.14, 0.74)**	small
**TW-H** [J ·kg^-1^]			** **	
Dominant	32.67 ± 6.59	30.47 ± 6.52	**0.33 (0.03, 0.63)**	small
Non-Dominant	31.75 ± 6.73	29.82 ± 6.02	**0.30 (0.00, 0.60)**	small
**H/Q Ratio**				
Dominant	0.59 ± 0.08	0.57 ± 0.06	0.27 (-0.02, 0.57)	small
Non-Dominant	0.58 ± 0.08	0.57 ± 0.06	0.14 (-0.16, 0.43)	trivial

*Note*: Effects sizes in bold significant difference between groups (p < 0.05), PT-Q–peak torque of quadriceps, PT-H–peak torque of hamstrings, H/Q–hamstring/quadriceps ratio, TW-Q–total work of quadriceps and TW-H–total work of hamstrings.

Table 2 shows mean inter-limb asymmetry data for all isokinetic strength measures. The junior group exhibited lower imbalances than the senior group for all conditions, apart from PT-Q, where similar average values were recorded. All these differences represented trivial ES values (*g* range = 0.00 to 0.15) and did not impact any strength measurements.

**Table 2:** Mean ± standard deviation inter-limb asymmetry data (%), Hedges g effect sizes with 95% confidence intervals (CI).

Asymmetry Variables	Senior Group n = 109	Junior Group n = 74	Hedges g (95% CI)	g Descriptor
**PT-Q**	6.42 ± 4.85	6.43 ± 5.48	0.00 (-0.30, 0.30)	trivial
**PT-H**	8.55 ± 6.16	7.98 ± 6.04	0.09 (-0.20, 0.39)	trivial
**TW-Q**	6.81 ± 4.79	6.45 ± 5.23	0.07 (-0.23, 0.37)	trivial
**TW-H**	9.69 ± 6.98	8.64 ± 6.72	0.15 (-0.15, 0.45)	trivial

*Note*: Effects sizes in bold significant difference between groups (p < 0.05), PT-Q–peak torque of quadriceps, PT-H–peak torque of hamstrings, TW-Q–total work of quadriceps and TW-H–total work of hamstrings.

**Fig 1:**
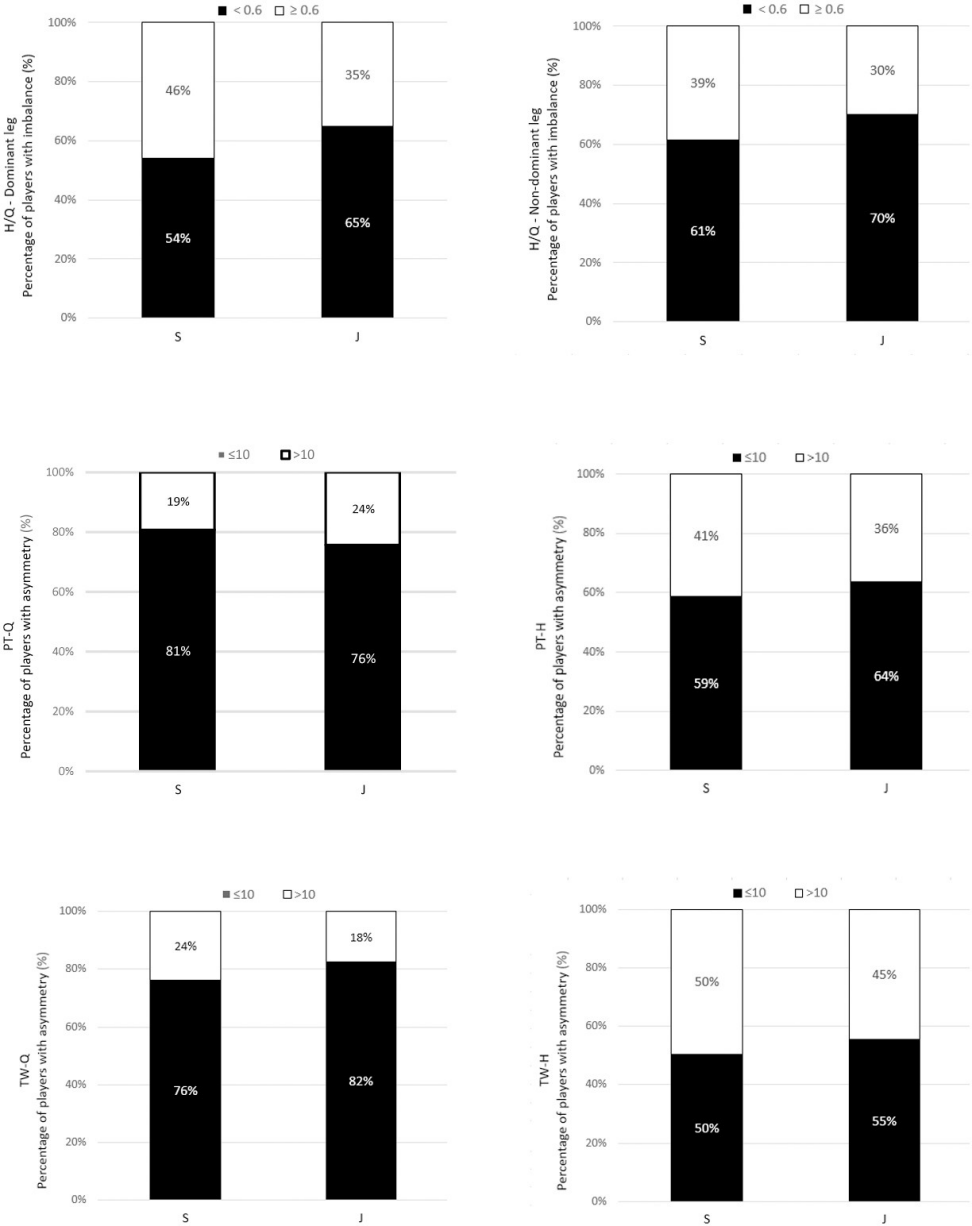
The percentage distribution of players with intra-limb imbalance (≥0.6) and inter-limb asymmetry (>10%) scores for both groups soccer players. S–senior, J–junior, PT-Q–peak torque of quadriceps and PT-H–peak torque of hamstrings. TW-Q–total work of quadriceps and TW-H–total work of hamstrings.

**Fig 2:**
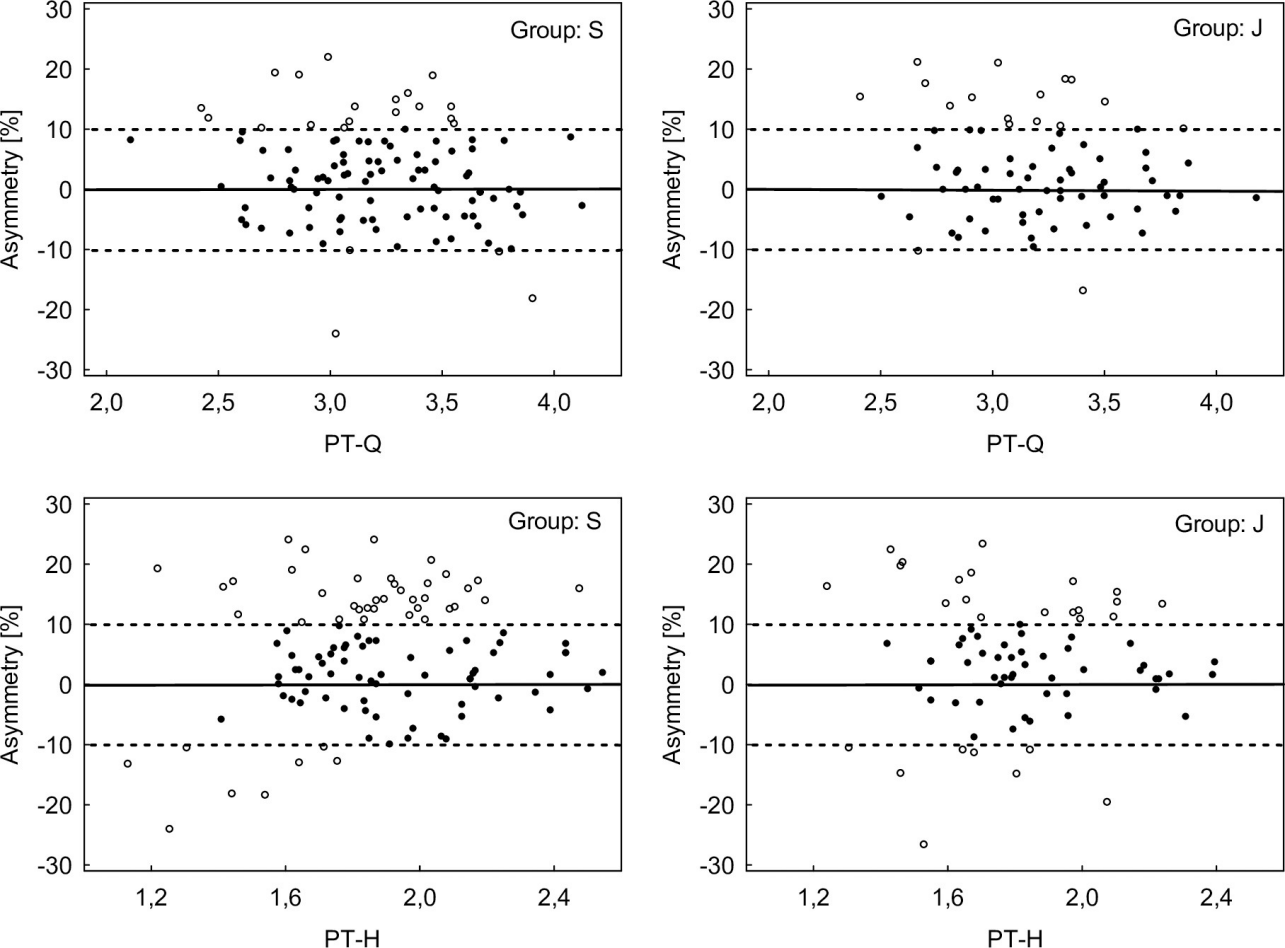
Individual magnitude and direction of inter-limb asymmetry for peak torque in both groups soccer players. Empty circles represent players with asymmetry > 10%. S–senior, J–junior, PT-Q–peak torque of quadriceps and PT-H–peak torque of hamstrings.

**Fig 3:**
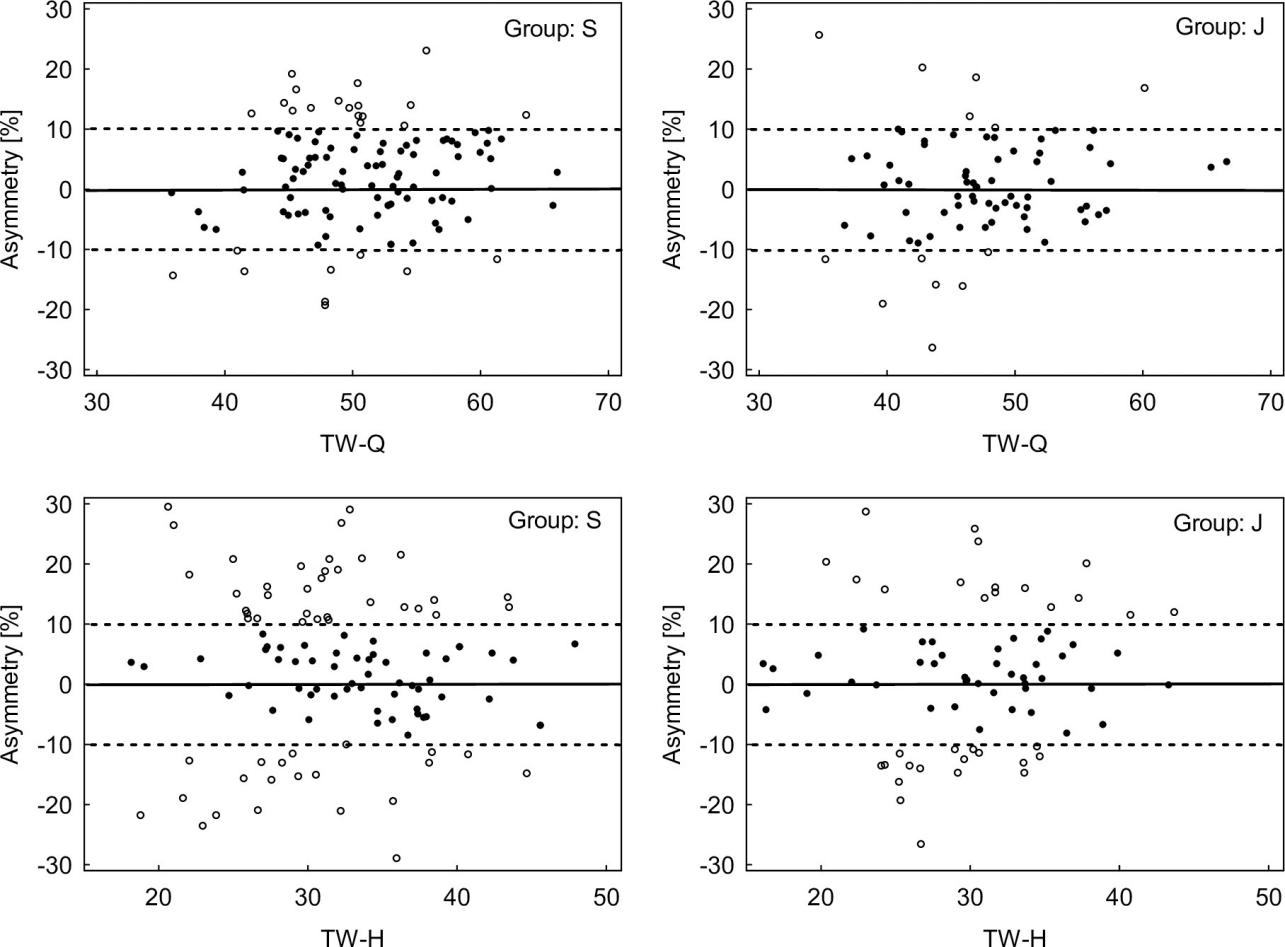
Individual magnitude and direction of inter-limb asymmetry for total work in both groups soccer players. Empty circles represent players with asymmetry > 10%. S–senior, J–junior, TW-Q–total work of quadriceps and TW-H–total work of hamstrings.

Fig 1 shows mean intra-limb imbalance data for H/Q ratio (≥0.6) with a higher proportion of these values in the senior group (46% for DL) and (39% for NDL) compared to the junior group (35% for DL and 30% for NDL, respectively). However, these differences were not influential (χ2 = 2.092, p > 0.05 for DL and χ2 = 1.502, p > 0.05 for NDL, respectively) and the strength of the relationship between the variables was low ([phi = 0.107] for DL and [phi = 0.091] for NDL, respectively). Fig 1 also shows mean inter-limb asymmetry (>10%) for all strength measures, with a higher percentage of these values in the senior group (41% for PT-H, 24% for TW-Q and 50% for TW-H) compared to the junior one (36%, 18% and 45%, respectively). The exception to this was PT-Q, which was higher in the junior group than the senior group (24% vs. 19%, respectively). However, these differences were not influential (χ2 = 0.672, p > 0.05 for PT-Q; χ2 = 0.425, p > 0.05 for PT-H; χ2 = 1.038, p > 0.05 for TW-Q and χ2 = 0.432, p > 0.05 for TW-H). The strength of the relationship between the variables was small and adequate (phi = 0.061) for PT-Q; (phi = 0.048) for PT-H; (phi = 0.075) for TW-Q and (phi = 0.049) for TW-H, respectively. The direction and magnitude of individual values for intra-limb asymmetry in both groups are shown in Figs 2 and 3.

## Discussion

The purpose of this study was twofold: (1) to assess the isokinetic strength exerted by the knee extensor and flexor muscles in top senior and junior soccer players; and (2) to identify the nature, and quantify the level of intra- and inter-limb strength imbalances and asymmetries between the both groups. It was hypothesized firstly that senior players will have a greater isokinetic strength exerted by both knee extensor and flexor muscles than their junior peers, and secondly than senior soccer players will demonstrate lower limb asymmetries and imbalances than junior players. Overall, the first hypothesis was only validated for the variables representing muscle work (TW-Q and TW-H), and not for the variables expressing the maximal strength (PT-Q and PT-H). The second hypothesis was invalidated.

As far as the authors are aware, this study was the first of its kind to investigate such a large cohort of elite senior and junior soccer players. The findings widen and deepen our understanding of the profiles of soccer players playing at the highest level of the Polish game (senior and junior). The main findings were: (1) no differences were noted between groups for PT-Q and PT-H of an angular velocity at 60° ·s^-1^, (2) senior players exhibited greater strength levels for TW-Q and TW-H of an angular velocity at 240° ·s^-1^, (3) no differences were observed between groups for intra-limb (H/Q) ratio, (4) overall, no intergroup differences were noted for inter-limb asymmetry data for all isokinetic strength measures, (5) lastly, no intergroup differences were observed for intra-limb imbalances and inter-limb asymmetry data for all isokinetic strength measures in relation to the “normative values” adopted in the literature, and which are used to identify athletes at greater risk of knee injury.

The present results show that elite senior players did not exhibit higher strength levels than elite junior players for all isokinetic variables. There were only differences between the groups for the TW-Q and TW-H variables in both legs. This is why the initial hypothesis could not be fully supported. These results concur with the findings of previous studies [12], which found that professional soccer players (PRO) did not show any differences at the level of body weight-related PT-Q and PT-H in both concentric and eccentric contraction modes, compared to U-21 and U-17 soccer players. A similar phenomenon was described by Eustace et al. [42], who reported an insignificant effect of age on isokinetic strength between PT of an elite senior group (age 25.09 ± 3.83 years) and a youth group (age 17.00 ± 0.06 years). Lehance et al.’s [12] and Eustace et al.’s [42] findings indicate that junior players achieve the maximum level of strength for both knee flexor and extensor muscles at approximately 17 years old. Likewise, research by Maly et al. [4] shows that soccer players aged approximately 17 did not show any differences in muscle strength (peak torque for flexor and extensor muscles) when compared to older (U-19 and U-21) cohorts. In contrast with these results, Bishop et al. [13] have shown in their recent study that professional players are stronger in both the quadriceps and hamstrings than academy (U-18) soccer players, in both slow (60°·s^-1^) and high (300°·s^-1^) velocity contractions. These results are again consistent with a recent study [43] that confirmed the same relationships by analyzing the same isokinetic strength variables at the same angular velocities and comparing professional soccer players with elite academy players (U-19). The age-related discrepancy in isokinetic muscle strength variables in soccer players may be due to the differences in factors such as players’ competition levels, testing period, training load, number of matches played, and strength training regimes [4,44].

Moreover, the present results reveal that the TW-Q and TW-H variables are higher for seniors than for juniors, meaning that seniors produce more work with quadriceps and hamstrings than juniors. In the context of an isokinetic muscular contraction, total work depicts the muscle’s ability to generate strength over the entire range of movement [44]. Over the same joint amplitude, seniors are thus able to produce greater work than juniors with the knee flexor and extensor muscles. As mentioned above, experienced soccer players (seniors) are likely to have developed neuromuscular properties as a result of high external training volume and a large number of matches played [24,31]. This enables them to maintain higher muscular strength until the end of the movement by continuing to recruit fibers for longer time than junior soccer players. Recent studies on soccer have indeed compared training and match loads between youth and adult players [45,46]. The study of Coppalle et al. [45] reported an accumulation of more matches and minutes played in matches among adult players compared to U19 players in in-season period. Thus, neuromuscular properties need to be taken into account in young players, due to incomplete muscle and skeletal development [13].

The present results also provide a comparative analysis of flexor-extensor strength imbalances (i.e., intra- limb asymmetry) and inter-limb asymmetry for each muscle group and strength variables between senior and junior players. They showed no differences for either H/Q ratios between groups in both legs (Table 1) with small to trivial ES values (*g* range = 0.14 to 0.27), and the inter-limb asymmetry for all strength measures (Table 2) represented trivial ES values in all cases (*g* range = 0.00 to 0.15), thus rejecting our second working hypothesis. Our findings were confirmed by the previously reported study of Lehance et al. [12], who also found no differences concerning H/Q imbalances or intra-limb asymmetries for all modes of contraction and angular velocities for the PRO, U-21 and U-17 groups. These findings align with a recent study by Parpa et al. [28], which showed no difference for inter-limb asymmetries between young (age = 17.20 ± 0.55 years) and adult (age = 28.23 ± 4.95 years) soccer players. Recent studies by Maly et al. [4] also confirm that the age of the surveyed soccer players in four age categories (U-16, U-17, U-19 and U-21) did not have an influence on intra-limb strength ratios or inter-limb strength asymmetries. However, it has been shown that professional senior players displayed greater H/Q ratio values for the dominant and non-dominant limbs at 60° ·s^-1^ (*g* range = 0.46–0.72), but not for the 300° ·s^-1^ condition (*g* range = -0.09–0.33), and lower inter-limb differences in the academy players for all conditions except the hamstrings at 60° ·s^-1^ (*g* = -0.23) [13]. Other relationships between inter-groups are presented in a study by Beato et al. [43], which indicates that professional players displayed a slightly higher H/Q ratio compared to elite academy players, while no difference in hamstrings and quadriceps inter-limb strength asymmetry was found between these groups of players. A similar phenomenon was described by Herdy et al. [47], who found differences for the conventional H/Q ratio in dominant leg between the adult professional and the U-17 soccer players. Overall, based on available data, it is currently difficult to establish a consensus on intra-limb imbalance and inter-limb asymmetry. Hence, only descriptive analyses of these imbalances and asymmetries are likely to provide additional practical insights.

This situation justifies descriptive and comparative analyses of the magnitude of the intra-limb imbalances and inter-limb strength asymmetries relative to the “normative values” accepted in the literature that increase the risk of knee injury [26,38–40] (Fig 1). Nevertheless, they showed no differences between groups for H/Q ratio (with a cut-off value ≥0.6), and the strength of the association between the variables was small (phi = 0,107 for DL and phi = 0,091 for NDL, respectively). There were also no differences for all individual intra-limb asymmetry variables (with a cut-off value >10%), with similarly small strength of association between variables (phi = 0.049 for PT-Q; phi = 0.049 for PT-H; phi = 0.075 for TW-Q and phi = 0.049 for TW-H, respectively). The presented results, along with findings from several other studies [5,12,29,36] imply the need for further research in determining the cut-off value for PT, in order to categorize the sample as symmetrical or asymmetrical in groups of adult and youth soccer players, around which there is a lively discussion, albeit one that has not yet developed a clear consensus.

Another noteworthy aspect is the considerable standard deviations observed for each individual variable regarding inter-limb asymmetry (Table 2). This in turn may account for the lack of difference in between-group asymmetry. According to Bishop et al. [13], these findings underscore the necessity of considering the reliability of individual variables when interpreting individual asymmetry score validities. Conducting individual analyses for each player provides insight into which asymmetry values favor the DL with positive asymmetry outcomes and which favor the NDL, as indicated by negative scores. As such, this shows how one limb may be preferred to another [19]. In this context, our study presents the individual magnitude and direction of inter-limb asymmetry for both groups (Figs 1 and 2). Notably, for PT these distributions indicate a preference for asymmetry in favor of the DL, whereas for TW they demonstrate a more symmetrical distribution of between-leg asymmetry. This individualized information appears pivotal for implementing targeted training interventions aimed at optimally reducing imbalances and asymmetries.

As always, there are some limitations to this study and interpretation of the results should proceed accordingly. Firstly, it should be noted that our evaluations were confined to concentric muscle contraction variables, overlooking potential insights obtainable from examining eccentric PT-H. The incorporation of eccentric PT-H measurements would possibly reveal additional relationships between concentric and eccentric PT-H, enabling the calculation of dynamic H/Q ratios, and thus allowing for a more robust muscle profile report augmenting the comprehensiveness of our muscle profile analysis. Secondly, our research methodology did not include a breakdown of players by field position. We believe that there can be specific relationships between the demands of playing position and asymmetries. Thirdly, the inclusion of a wider age range of young players, e.g. before and after PHV (peak height velocity), would provide a broad spectrum for assessing asymmetries in the ontogenetic developmental process against adult players. Finally, caution is needed when interpreting these data, as the thresholds were applied arbitrarily). More broadly, in order to improve training for soccer players at all levels, the authors recommend that subsequent research should investigate the interactions between different elements of asymmetries, injuries and overall fitness.

## Conclusions

This study provides important comparative analyses of isokinetic strength performance and intra-limb imbalances, as well as inter-limb asymmetries in top senior and junior soccer players. The results showed a similar maximum strength capacity in both groups and higher muscle work in the senior group. No observed differences between the groups for intra-limb ratio and for inter-limb asymmetry overall and in relation to the “normative values” accepted in the literature that increase the risk of knee injury. This study demonstrates that isokinetic assessment provides a holistic picture of the imbalances/asymmetries. Strength and conditioning practitioners may use the results of the present study to prepare youth soccer players to the senior levels and also develop of injury prevention strategies. Finally, it is recommended that both mean and individual data in player profiles be collected in order to allow practitioners to better understand asymmetry.

## Supporting information

S1 File Assessments database. (XLSX)
